# Real-time detection of spoken speech from unlabeled ECoG signals: a pilot study with an ALS participant

**DOI:** 10.1088/1741-2552/ae0965

**Published:** 2025-10-06

**Authors:** Miguel Angrick, Shiyu Luo, Qinwan Rabbani, Shreya Joshi, Daniel N Candrea, Griffin W Milsap, Chad R Gordon, Kathryn Rosenblatt, Lora Clawson, Nicholas Maragakis, Francesco V Tenore, Matthew S Fifer, Nick F Ramsey, Nathan E Crone

**Affiliations:** 1 Department of Neurology, The Johns Hopkins University School of Medicine, Baltimore, MD, United States of America; 2 Department of Biomedical Engineering, The Johns Hopkins University School of Medicine, Baltimore, MD, United States of America; 3 Department of Electrical and Computer Engineering, The Johns Hopkins University, Baltimore, MD, United States of America; 4 Department of Computer Science, The Johns Hopkins University, Baltimore, MD, United States of America; 5 Department of Cognitive Science, The Johns Hopkins University, Baltimore, MD, United States of America; 6 Research and Exploratory Development Department, Johns Hopkins Applied Physics Laboratory, Laurel, MD, United States of America; 7 Departments of Plastic and Reconstructive Surgery & Neurosurgery, The Johns Hopkins University School of Medicine, Baltimore, MD, United States of America; 8 Department of Anesthesiology & Critical Care Medicine, The Johns Hopkins University School of Medicine, Baltimore, MD, United States of America; 9 UMC Utrecht Brain Center, Department of Neurology and Neurosurgery, University Medical Center Utrecht, Utrecht, The Netherlands

**Keywords:** brain-computer interface, voice activity detection, electrocorticography

## Abstract

*Objective*. Brain–computer interfaces hold significant promise for restoring communication in individuals with partial or complete loss of the ability to speak due to paralysis from amyotrophic lateral sclerosis (ALS), brainstem stroke, and other neurological disorders. Many of the approaches to speech decoding reported in the BCI literature have required time-aligned target representations to allow successful training—a major challenge when translating such approaches to people who have already lost their voice. *Approach*. In this pilot study, we made a first step toward scenarios in which no ground truth is available. We utilized a graph-based clustering approach to identify temporal segments of speech production from electrocorticographic (ECoG) signals alone. We then used the estimated speech segments to train a voice activity detection (VAD) model using only ECoG signals. We evaluated our approach using a leave-one-day-out cross-validation on open-loop recordings of a single dysarthric clinical trial participant living with ALS, and we compared the resulting performance to previous solutions trained with ground truth acoustic voice recordings. *Main results*. Our approach achieves a median timing error of around 530 ms with respect to the actual spoken speech. Embedded into a real-time BCI, our approach is capable of providing VAD results with a latency of only 10 ms. *Significance*. To the best of our knowledge, our results show for the first time that speech activity can be predicted purely from unlabeled ECoG signals, a crucial step toward individuals who cannot provide this information anymore due to their neurological condition, such as patients with locked-in syndrome. *Clinical Trial Information*. ClinicalTrials.gov, registration number NCT03567213.

## 1. Introduction

Several neurological disorders, including amyotrophic lateral sclerosis (ALS), can result in severe paralysis and loss of speech, having devastating effects on the quality of life of affected individuals. Recent advances in implantable brain–computer interfaces (BCIs) have raised hope for the restoration of communication in this clinical population [1, 2] by utilizing neural activity acquired directly from the cerebral cortex to control a neuroprosthetic device that produces text [3–7] or synthesizes speech [7–11]. Those BCIs are currently trained using supervised learning paradigms where neural activity is mapped onto target representations [12, 13], such as phonemes or acoustic units, and are therefore dependent on accurate temporal alignments to achieve proper outputs. For this reason, many prior studies in the field have relied on datasets collected from patients who had normal speaking capabilities, such as epilepsy patients [8, 11, 14, 15] or patients who underwent surgery for glioma removal [16, 17]—datasets where the temporal alignment can be obtained from simultaneous acoustic recordings.

In recent years, clinical trials have begun exploring the extent to which approaches previously used in normal speaking subjects can be translated to people in actual need for such a technology [3, 7, 18, 19], and while those enrolled clinical-trial participants were speech impaired, their diseases had not yet been progressed into a state of total paralysis that prevented inferring such an alignment. However, in cases where the disease has already progressed to the locked-in syndrome (LIS) [20, 21], it may not be possible to infer the temporal alignment at all from acoustic data. In pioneering work by Guenther *et al* [22], a participant living with LIS was able to accurately synthesize vowels continuously using a Kalman filter-based decoding approach with closed-loop neurofeedback. Additionally, more recent work by Chaudhary *et al* gave a completely locked-in patient a novel means of communications by spelling sentences using a paradigm that required modulating firing rates with respect to auditory feedback [23].

In this study, we present a technique for acoustic-free model training by assuming that no temporal alignment can be obtained from simultaneous microphone recordings. For this early work, we focus only on localizing and identifying neural activity related to speech processes. Voice activity detection (VAD) systems play a crucial role in acoustic speech processing fields, such as automatic speech recognition [24] or speaker diarization [25], where they may be used in early processing stages to exclude non-speech data when computing acoustic features or embedding vectors. Similarly, many recent BCI studies have also utilized approaches to locate and isolate neural activity related to speech production in their pipelines as an intermediary step to constrain the solution space of speech decoding tasks, both for word recognition [19, 26] and synthesis applications [18]. Another application for these neural VAD (nVADs) systems of particular relevance to BCIs is to prevent leakage of speech-related activity into computation of baseline statistics within real-time systems. Decoding performance can degrade over time because the feature space may shift linearly beyond the range expected from the training data. nVAD techniques could help here to determine which parts of the neural data should be considered when updating a running baseline, rather than relying only on a fixed time window containing both speech and non-speech activity. Previous methods reported in the literature predominantly relied on supervised learning machines trained directly on acoustic ground truth [18, 19, 27] or labeled information [26] inferred from behavioral cues [28]. Such approaches may be difficult to use in individuals who are unable to vocalize or produce any observable articulatory movements. In contrast to these supervised methods, Wang *et al* presented results on decoding human behavior, including speech, completely in an unsupervised manner using hierarchical clustering on power spectral ECoG features at a resolution of two minute segments [29].

Here, we present proof of concept results on unsupervised detection of neural voice activity from unlabeled ECoG signals at a temporal resolution applicable for real-time decoding. We did so by setting up an experiment in which a clinical trial participant was instructed to read single words, and where the majority of time for each recording session did not carry speech activity—a design decision we actively exploited to automatically assign identified segments as either speech or non-speech classes. We utilized a graph-based clustering approach [30] to find structural patterns with a fixed temporal context in high-gamma (HG) activity extracted from ECoG recordings, and used those estimated clustering labels to train three classification models previously used in the literature for nVAD tasks, namely a recurrent neural network (RNN), a convolutional neural network (CNN) and logistic regression (LR). In our evaluation, we first quantified the alignment error between estimated labels from the clustering approach and ground truth acoustic speech information to determine ranges of expected error rates. Next, we compared the performance of our three classifiers trained on those estimated labels with respect to baseline models trained on VAD labels inferred from acoustic speech. From here, we then inspected how well our model translated to unseen words. Eventually, we checked the real-time performance with our trained model in a playback scenario to ensure our approach can provide nVAD predictions within an online system.

## 2. Material and methods

### 2.1. Participant and experiment design

**Figure 1.**
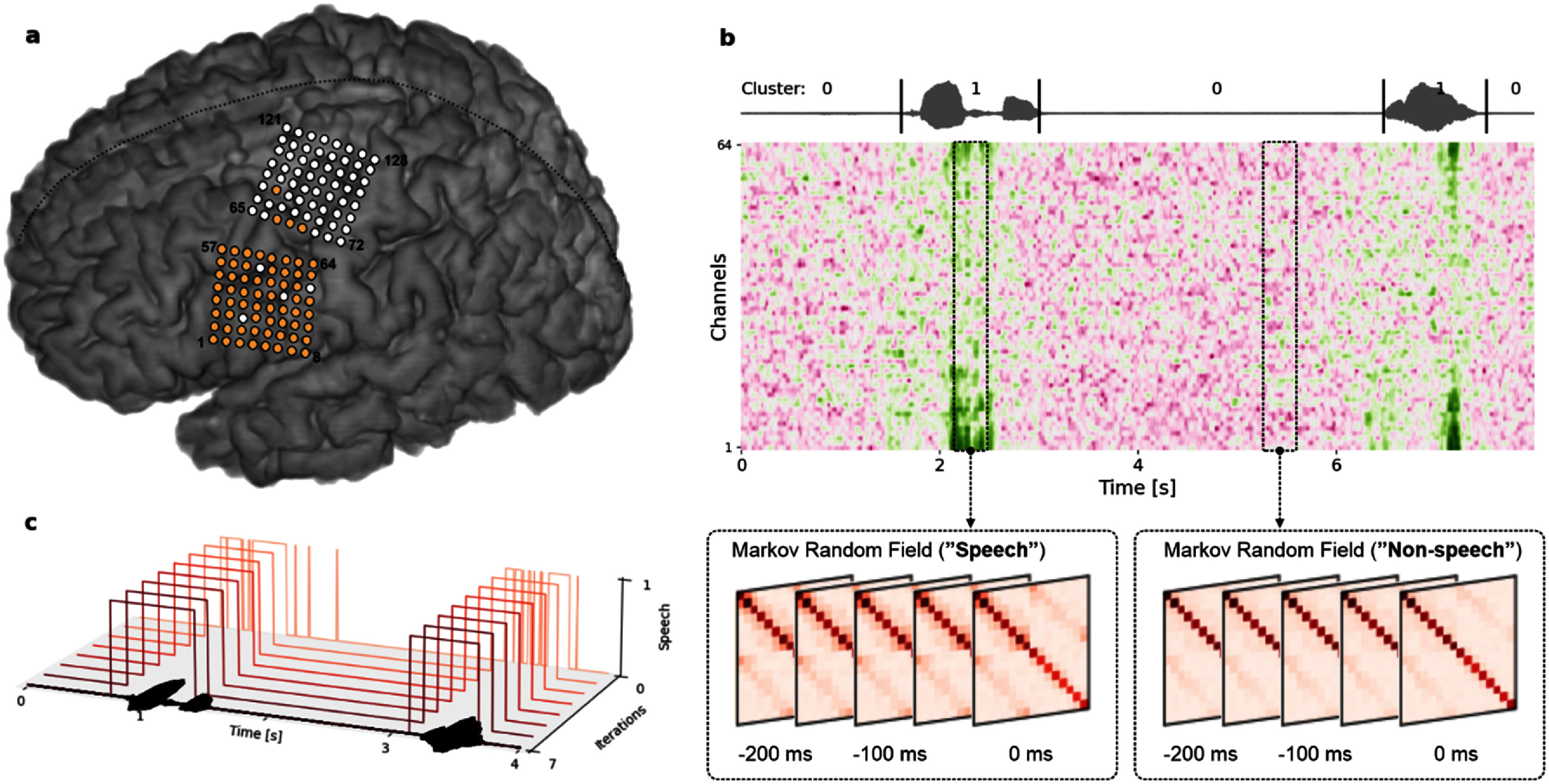
Overview of the experiment setup and clustering approach. (a) Placement of implanted electrode grids covering speech and upper limb cortical regions. Electrodes highlighted in orange were selected for this study based on previous reported results [18]. (b) Illustration of the TICC clustering approach to identify speech and non-speech segments in each trial using one Markov random field per cluster. The gray line represents the ground truth acoustic speech signal, time-aligned with the corresponding high-gamma activity. Cluster assignments for 0 (non-speech) and 1 (speech) where calculated based on voice activity detection from the acoustic signal [45]. Panels for both Markov random fields emphasize internal parameter matrices after training. (c) Visualization of the iterative clustering process of the TICC algorithm, starting from an initial alignment derived from Gaussian mixture clustering, until convergence. The acoustic waveform on the *x*-axis serves as a reference to the found speech clusters.

We conducted an experiment with a clinical trial participant (CC01, 62 years old, male) with dysarthria due to ALS, who had been implanted with two ECoG arrays with 64 electrodes each (4 mm center-to-center spacing, 2 mm diameter) covering speech and upper-limb cortical areas (figure 1(a)). The participant could speak, but his speaking capabilities were limited, and continuous speech was mostly unintelligible due to his neurological condition [18, 19] (speech was rated with 1 point out of 5 on the ALSFRS-R measure [31]). In a speech production task, we presented single words on a monitor in front of him and gave instructions to read them out loud. For each trial, the target word was presented for 2 s following an inter-trial interval of 3 s. Overall, the word pool consisted of 50 words [3], and each word was repeated twice in each session. We repeated this experiment across 10 d over a period of 9 weeks. Furthermore, we also collected single word data from a larger word pool of 688 words, which we used to quantify generalization towards unseen words. In this corpus, each word only appeared once, and none appeared in the training data. At the start of each recording day, we conducted a syllable repetition task, which was used for normalizing the neural data. The syllable repetition task was constant across all days to achieve similar statistics for the baseline, in accordance with a prior publication with the same study participant [18].

Neural data was digitized using a NeuroPort System (Blackrock Neurotech, Salt Lake City, UT, USA) with a sampling rate of 1 kHz. Audio data was recorded at 48 kHz using an external microphone (BETA^®^ 58 A, SHURE, Niles, IL). We used BCI2000 [32] for stimulus presentation and for aligning neural and acoustic signals for offline analysis. The clinical trial (ClinicalTrials.gov, NCT03567213) was approved by the Johns Hopkins University institutional review board and by the FDA (under an investigational device exemption) to test the safety and preliminary efficacy of a brain-computer interface composed of subdural electrodes and a percutaneous connection to external EEG amplifiers and computers. This study was conducted in accordance with the Declaration of Helsinki and all local statutory requirements. The participant gave informed consent after being counseled about the nature of the research and implant-related risks, and was implanted with the study device in July 2022.

### 2.2. Cortical mapping

The positioning of both subdural ECoG grids was determined via anatomical landmarks from pre-operative structural (MRI) and functional imaging. After the surgical implantation of the grids, we conducted a post-operative CT scan, which was co-registered to a pre-operative MRI for verification of the anatomical locations of the two grids. Figure 1(a) shows a rendering of the participant’s brain and the locations of both electrode grids, where the 64 electrodes highlighted in orange were relevant in this study with respect to prior observations [18] about encoded speech activity.

### 2.3. Signal processing and feature extraction

We obtained speech-related features from raw ECoG signals by extracting the HG band, which has shown to track closely the location and timing of speech production neural activation [33, 34] and has been successfully employed in previous studies for speech BCIs [4, 18, 19, 35–37].

First, we removed all bad channels (19, 38, 48 and 52) based on visual inspection and applied a common average referencing filter across each grid independently. Next, we selected the top 64 channels with the strongest activation during overt speech production, identified in a previous study [18] with the same clinical trial participant. We then used a bandpass filter (IIR Butterworth, 4th order) to extract the broadband HG band in the range of 70–170 Hz and a notch filter (IIR Butterworth, 4th order) to attenuate the first harmonic of the line noise in the range of 118–122 Hz. Finally, for each channel we computed logarithmic power features with respect to a window size of 50 ms and a frame shift of 10 ms. We normalized all features to zero mean unit variance (z-score normalization) with respect to a syllable repetition task conducted at the beginning of each recording day to calibrate the system for day-specific HG changes (see supplementary figure S1 about the stability of the ECoG signals during the study period). Moreover, we added one more step to prepare the features for classifiers that do not model temporal dependencies. In those cases, we augmented each frame with non-overlapping frames up to 300 ms into the past. Since every frame was calculated on 50 ms windows, this resulted in a context stacking of seven frames (0 ms, −50 ms, −100 ms, −150 ms, −200 ms, −250 ms, and −300 ms). This step was not included in the clustering procedure as the clustering algorithm itself manages a fixed window of past frames through multiple layers in the Markov random field (MRF) to account for the temporal relationships in each cluster.

The acoustic data for performance evaluation was collected at 48 kHz, resampled to 16 kHz and segmented into corresponding windows of 50 ms and 10 ms frameshift to match the alignment with the HG features. We verified that no channels had been contaminated with acoustic artifacts by using Roussel’s method [38]. The details of the contamination report are given in supplementary figure S2.

### 2.4. Unsupervised temporal localization of speech production

Time series clustering is an active field of research in knowledge discovery and data mining communities [39]. In contrast to conventional clustering methods such as *K*-Means or Gaussian Mixture Models, which consider individual data points independently from their temporal context, sub-sequence time series clustering algorithms are designed to identify common patterns that are distinct across different sub-sequences within the same data [40]. These approaches are based on variations of the dynamic time warping algorithm [41, 42], mixtures of autoregressive moving averages [43], or data encoding techniques to determine meaningful cluster centers [44], among others.

To identify speech-associated activity in neural recordings, we adopted a graph-based clustering approach named Toeplitz Inverse Covariance-based Clustering (TICC) [30], specifically designed for discovering a predefined number of common subsequences in multivariate time series data while preserving the temporal dependencies within each cluster. This unsupervised algorithm defines one MRF per cluster and describes relationships in the form of connections between input features. In our study, these connections would describe dependencies between the neural activity of different electrodes, both with respect to spatial and (potentially) causal temporal patterns.

TICC’s training procedure is based on a variation of the expectation maximization algorithm to alternate between assigning points to clusters and updating the MRFs’ parameters. Here, the assignment step (E) utilizes the current state of the MRFs to compute the likelihood of each HG frame with respect to each cluster, while also taking the temporal consistency into account to assign neighboring frames to the same cluster. The cluster assignment step results in the path with the minimum cost, obtained using a dynamic programming paradigm. Once this path has been found, the maximization step (M) updates the cluster parameters of each MRF based on their assigned data points, while ensuring that the Toeplitz constraint is not violated. The TICC algorithm repeats these two alternating steps until convergence when no data points are assigned to a different cluster anymore and are therefore stationary, which is guaranteed due to the convex nature of the maximization step.

Besides the number of clusters, the TICC algorithm can be configured with respect to the length of the temporal context and regularization parameters. By specifying multiple layers for the MRFs, data points will not get clustered in isolation but in context to neighboring past observations, allowing it to learn cross-time relationships. Note that temporal layers in the MRFs also obey the Toeplitz constraint to be time-invariant. The regularization parameters *β* and *λ* signify the penalty factor for adjacent subsequences being assigned to the same cluster and denote the sparsity level in the MRF’s graph structure characterizing each cluster, respectively. A higher *β* value will result in a greater likelihood of adjacent subsequences being assigned to the same cluster. In the case of single layer MRFs with *β* = 0, each frame—including their context for multi layer MRFs—gets assigned to a cluster independently of all other frames.

Figure 1(b) shows an illustration of the TICC clustering approach. Two MRFs segment the HG activity into speech and non-speech classes. In this example, both MRFs have multiple layers to not only draw insights from spatial characteristics but also capture temporal dynamics of up to 200 ms into the past. The gray waveform at the top has been time-aligned to the neural recordings for visual attribution of the HG activity. Although the clustering assignments do not reveal which clusters belong to speech activity due to their unsupervised nature, we can infer cluster classes based on the length of their subsequences—exploiting the setup of the experiment design. Figure 1(c) visualizes the clustering process for one recording session. The *x*-axis represents time and shows a snippet of two trials and the acoustic speech signal as a reference guide. The *z*-axis shows each iteration from the TICC algorithm until convergence, where found cluster alignments are plotted as curves. The *y*-axis indicates found speech activity. We based our initial alignments (iteration 0) on clusters found by a Gaussian mixture model, and iteratively optimized those using the TICC algorithm.

In order to infer suitable hyperparameters for the TICC algorithm we utilized ECoG recordings from a single patient with drug-resistant epilepsy (17 years old, male) who had undergone video-EEG monitoring to localize his seizure onset zone. We particularly chose this data as the implanted ECoG grid covered cortical speech areas similar to our clinical trial participant (see supplementary figure S3 for details about the grid placement in the epilepsy surgery patient). Note that the electrode grid in the epilepsy surgery patient was implanted in the right hemisphere, yet we were able to measure strong speech-related high gamma activity during speech production. Similar observations have previously been reported in the literature [13]. We ran a grid search across predefined ranges for the *β* and *λ* hyperparameters and selected those which achieved lowest alignment errors with respect to ground truth voice activity of the epilepsy patient, leading to a hyperparameter configuration of *β* = 50 and *λ* = 11 × 10^−4^.

### 2.5. Neural voice activity detection approaches

We based and evaluated the nVAD on three classifiers, namely CNNs similar to the LeNet architecture [27, 46], LR with L1 regularization, and the same RNN architecture from our previous study on synthesizing speech online [18], originally inspired by the work from Zen and Sak [47].

The LeNet-like model is based on a sequential network architecture, consisting of two convolutional and two max pooling layers in the feature extraction part, where each pooling layer follows directly its convolutional layer. Both convolutional layers use a kernel size of 3 and increase the number of feature maps by 32. The pooling layers are configured with a kernel size and stride of 2. Following the feature extraction part, we use three fully-connected layers with 128 and 64 hidden units as well as 10 output units. Moreover, we utilized tanh as a non-linear activation function throughout the network architecture and softmax at the output layer. For the training configuration, we employed Adam [48] with a learning rate of 0.0001 and limited network training to 10 epochs.

For the RNN, all recurrent layers utilize long-short term memory cells [49] to learn the temporal dynamics across the individual channels. In total, the network architecture comprises three layers: two LSTM layers with 100 units each and one linear layer with 2 output units, resulting in 231 682 internal weight parameters. Similar to the CNN, we used the cross-entropy loss in conjunction with the softmax activation function to estimate the error between network predictions and target labels during network training, and employed Adam as our optimizer with a learning rate of 0.0003 and trained the architecture for 20 epochs in each fold, while storing the best performing weights in accordance to the minimum validation loss. Network training uses the truncated backpropagation through time algorithm [50] with hyperparameters *k*_1_ and *k*_2_ set to 50 frames of HG activity, respectively, such that the unfolding procedure was limited to 50 frames (0.5 s) and repeated every 50 frames (0.5 ms).

### 2.6. Channel contribution analysis

The graphical dependency structures underlying cluster representations allow learned relationships to be interpreted and pinpointed to cortical areas known to elicit activity during speech—enabling us to verify that proper representations have been learned. The internal weight parameters of each MRF are arranged in the form of an adjacency matrix and encode the conditional dependencies between individual electrodes. Every connection with a nonzero weight indicates a relationship between those electrodes, where the sparsity of the adjacency matrix is controlled by the *λ* hyperparameter from the TICC algorithm. For this analysis, we calculated the absolute differences of those direct dependencies between both speech and non-speech MRFs’ parameters to reveal which connections between electrodes contribute to what extent to the decision-making process, further referred to as channel contributions when these dependencies were linked to the same electrode or were summed across all other electrodes for the total interdependency. Those differences can be mapped back and visualized on the implanted electrode grids, revealing which cortical areas and their corresponding differences in HG activity were identified by the clustering algorithm to distinguish speech and non-speech segments.

### 2.7. Evaluation metrics

We based our evaluation on the Levenshtein distance to determine the alignment error by computing the minimum amount of corrections that are necessary to change our estimated nVAD labels into those from the ground truth acoustic VAD. Since our approach operates at a precision of 10 ms frames, we assigned a respective cost of 10 ms to all *replace, insert* and *delete* operations within the Levenshtein distance so that the accumulated amount represents the total alignment error in milliseconds:

\begin{equation*}{\text{alignment}}{\text{ }}{\text{error}}\left( {p,{\text{ }}t} \right) = 10 \cdot \mathbb{D}\left( {p,{\text{ }}t} \right)\end{equation*}

where $\mathbb{D}$ is the Levenshtein distance, $p$ the prediction and $t$ the target. We particularly chose the Levenshtein distance in favor of the accuracy metric to provide a more accurate depiction of the misalignment by also accounting for temporal shifts in the prediction.

Moreover, we also report speech detection and false alarm probabilities, two metrics commonly used in acoustic voice activity detection [51, 52]. Here, the speech detection probability refers to the likelihood that a 10 ms frame containing speech information is correctly classified as speech. On the contrary, the false alarm probability refers to the likelihood that a frame gets classified as speech while the ground truth data contains no speech activity. While we used the alignment error based on the Levenshtein distance to measure overall classification performance of our approach, the speech detection and false alarm probabilities offer further insights into the tradeoffs between correctly identifying speech and avoiding misclassifications of non-speech segments.

### 2.8. Real-time system design

We built a real-time BCI that communicates directly with BCI2000 about any segments identified as speech. This system was implemented on top of *ezmsg* [53]—a Python framework that facilitates the development of closed-loop streaming applications by enforcing a software architecture composed of a directed acyclic graph structure. Each node in this graph is responsible for a particular self-contained task, such as computing HG features from raw ECoG signals. We used a network of such nodes to perform tasks that receive ECoG signals, compute features, predict voice activity and communicate results back to BCI2000, including logging functionality between all mentioned nodes for evaluation. In the backend, *ezmsg* utilizes asynchronous coroutines to enable concurrent executions of those tasks. Communication with BCI2000 was based on ZeroMQ as a networking abstraction layer. Our real-time processing pipeline could produce low-latency feedback as the accumulated computational cost per frame across all processing units (including scheduling of the asynchronous coroutines) resulted in 0.6 ms in mean with a standard deviation of 0.2 ms. This low computational cost enables the method to be used as a preprocessing step in more complex speech BCI systems.

## 3. Results

### 3.1. Identification of speech segments

**Figure 2.**
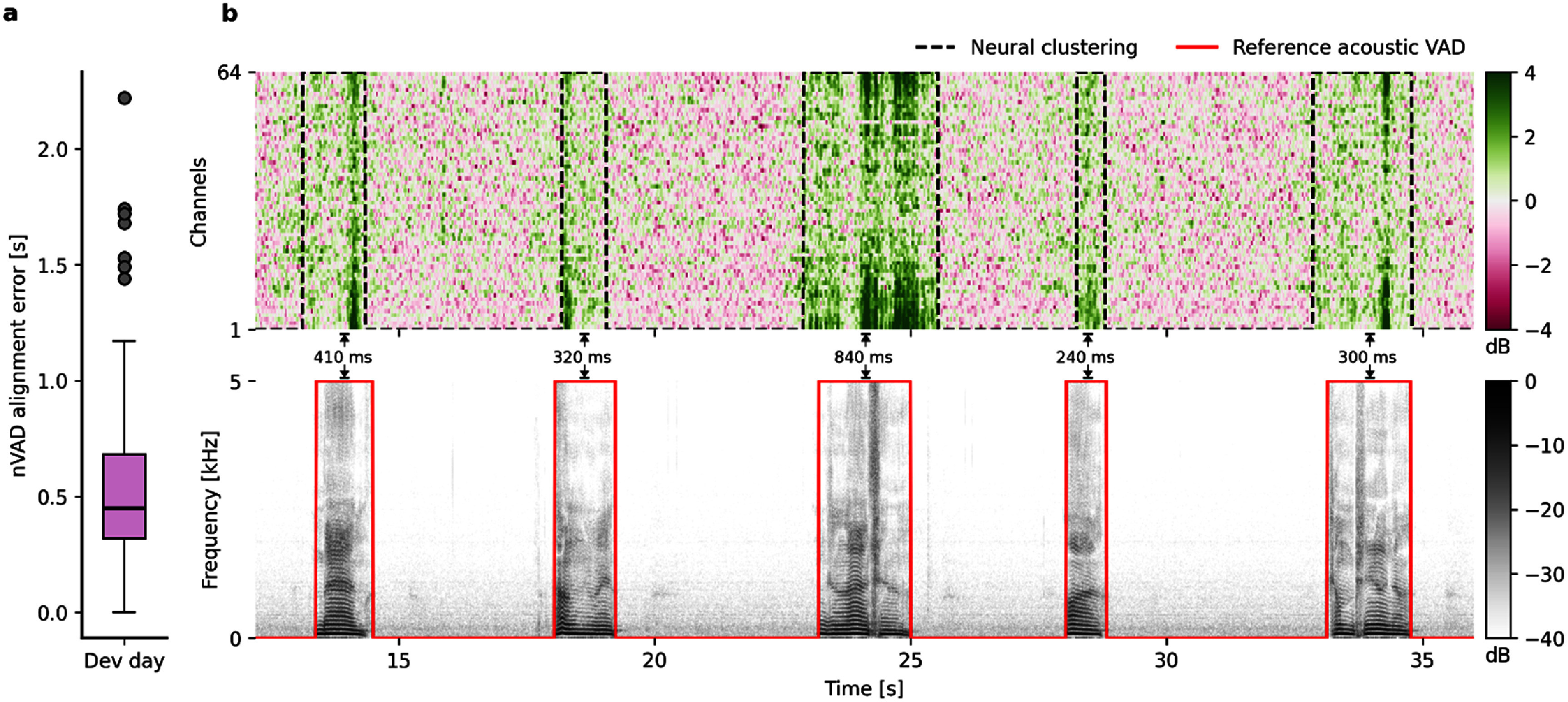
Comparison between VAD labels estimated from acoustic and high-gamma representations. (a) Minimum alignment error computed via Levenshtein distance between neural speech clusters and acoustic reference VAD using the development set (*n* = 204 trials). Box indicates boundaries between quartiles Q1 and Q3, and whiskers represent range of data within 1.5 times the interquartile range. Outliers correspond to trials for which no speech clusters could be found from the neural activity. (b) Visual example of the first 5 trials from the first day in the training set. Top panel shows estimated speech clusters using the TICC algorithm (dashed black line) on high-gamma features and bottom panel the corresponding time-aligned speech spectrogram from the acoustics with reference VAD (solid red line). The temporal values between the estimated speech clusters and the reference VADs indicate the specific alignment errors for these trials.

In this study, we only distinguished between speech and non-speech segments in the neural data, so that all words were summarized in one speech cluster. Another potential approach would be to cluster for each stimulus individually, assuming they were known upfront from the experiment design. However, preliminary empirical analyses suggested that resulting clusters per word find less accurate cluster assignments, potentially converging towards clusters that only identify part of speech segments. We hypothesize that this is related to the inherently smaller amount of data and less variability in the neural activity. When clustering for all words, it is not required to know a specific stimulus or the number of stimuli in advance and is thus also suitable for experiment designs where open questions are asked. We obtained ground truth voice activity information from time-aligned acoustic spectrograms of the microphone recordings which were only used to quantify the accuracy of identified speech segments. Our results are summarized in figure 2(a). On a held-out day solely used to report intermediate results from the clustering algorithm (from now on referred to as development set), we achieved a median alignment error of 450 ms per trial, while 75% of the trials were below 680 ms (average speech duration: 1.2 s). Speech detection and false alarm probabilities were measured at 0.74 and 0.08, respectively. In 13 out of 204 trials, speech could not be detected through the clustering approach and, additionally, 7 trials resulted in alignment errors above the average speech duration of 1.2 s. We considered a trial as not being detected if not a single frame from the ground truth has been identified as speech by our approach. Further investigations into those outliers revealed that primarily two types of sources were driving high alignment errors: (1) Speech was detected by the nVAD system, but at the wrong time where no ground truth data indicated speech presence, and (2) our system detected another speech segment in addition to the actual one. Figure 2(b) shows an excerpt of the first 5 trials of the first day in the training set for visual inspection. The top panel visualizes HG activity and how frames have been clustered after applying the TICC algorithm with the same configuration of hyperparameters obtained from the epilepsy patients data. The bottom panel shows the time-aligned speech spectrogram and ground truth VAD information based on the acoustic signal. Overall, the clustering approach can identify consecutive segments of spoken speech reliably in the majority of the cases, leading to labels that can be utilized to train a supervised model that predicts speech activity for an incoming stream of HG frames without calculating the minimum alignment path using dynamic programming strategies.

### 3.2. Temporal context provides less accurate speech clusters

Next, we analyzed if nVAD labels can be more accurately determined by including causal temporal contextual information. Here, we adapted the TICC algorithm to avoid repetitive information from the 40 ms overlaps in the feature extraction pipeline by adding a dilation hyperparameter indicating the spacing between consecutive HG frames. In accordance with Soroush *et al* [27], we investigated temporal dynamics up to 300 ms into the past. MRFs with only one layer correspond to no context information, with five layers up to 200 ms into the past (as represented in figure 1(b)), and with 7 layers of up to 300 ms, where each additional layer introduces a dilation of 5 frames to avoid repetitive information from the 40 ms overlap in the feature extraction pipeline.

**Figure 3.**
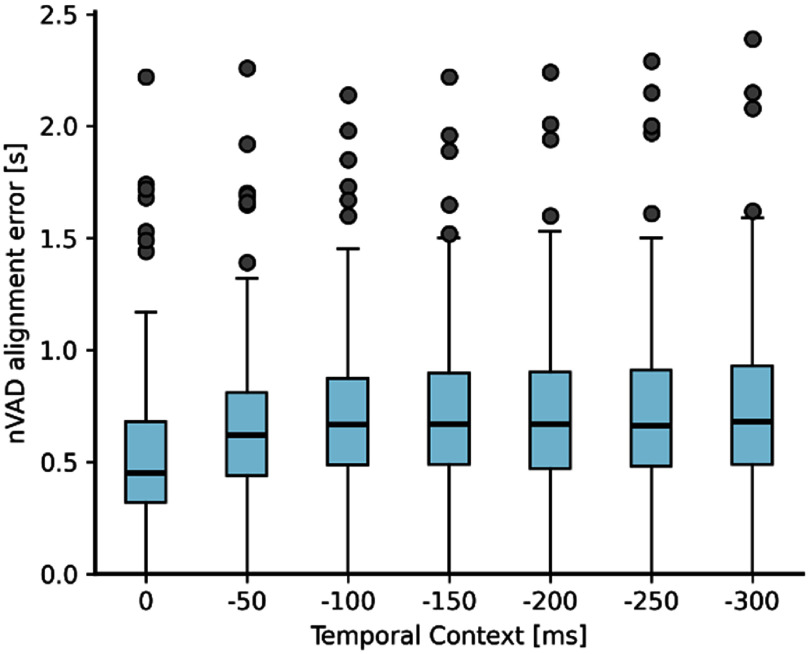
Adding temporal context leads to less accurate nVAD labels. Trend of inaccuracies for estimated nVAD labels increases with including context information. No context information and 300 ms into the past correspond to MRF’s with number of layers of 1 and 7, respectively. Evaluation was conducted on the development set to report the impact of including temporal context, however, we based our decision on using only one layer in the MRFs on the lowest error score obtained by the TICC algorithm with respect to the data from the epilepsy surgery patient. Boxes represent data between the quartiles Q1 and Q2 (*n* = 204 trials), while whiskers represent range within 1.5 times the interquartile range.

Similar to figure 2(a), we report our observations on the development set and used the minimum distance between estimated VAD labels and ground truth labels calculated on the speech spectrogram as the error metric, again with a cost of 10 ms per off-diagonal step in the alignment matrix. Figure 3 visualizes our results in the form of boxplots. We found that the median alignment error increased as more temporal context information was captured in each feature vector. We hypothesize that this trend stemmed from the growing number of features enabling more complex relationships in the spatio-temporal connections, which were inadequately supported by the limited amount of data, leading to increasingly inaccurate cluster parameters. We observed similar results with respect to the data used to determine appropriate hyperparameter choices; therefore, all further analyses were conducted with only one-layer MRFs.

### 3.3. Anatomic contributions from speech-related activity in motor cortices

**Figure 4.**
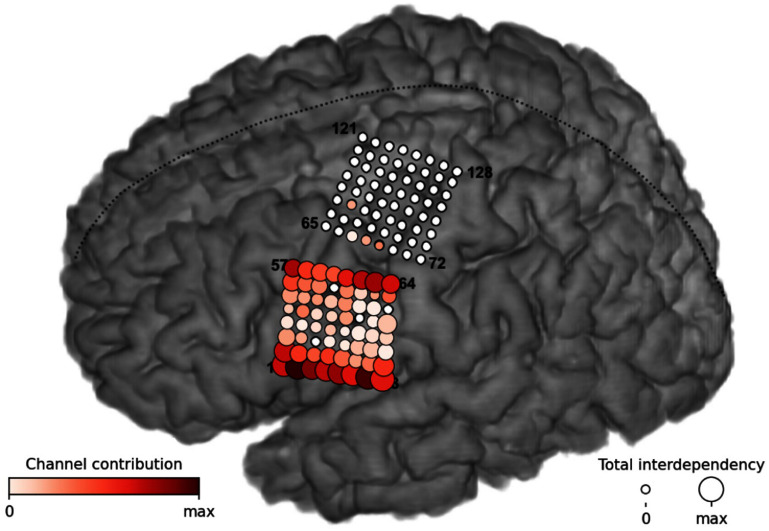
Cluster assignments mainly driven by differences in inter-electrode connections in vM1 and dM1. Visualization of the differences in the found Markov random field structures between both speech and non-speech clusters. The color coding of the circles represents electrode contributions, while the size indicates the strength of inter-channel dependencies. These relationships show that the TICC algorithm focused primarily on spatial high-gamma activity patterns between electrodes in ventral (vM1) and dorsal (dM1) parts of the primary motor cortex when deciding which cluster to assign.

We applied the steps outlined in the channel contribution analysis section to interpret the differences in learned relationships between speech and non-speech clusters. Our findings are visualized in figure 4. Each circle on the brain plot belongs to one channel. The color of the circle represents how much a particular channel contributed in the decision-making process of the clustering assignment and the size indicates the total sum of the interdependencies between channels. The plot reveals that the differences in HG activity features from electrode channels located in ventral (vM1) and dorsal (dM1) parts of the primary motor cortex were predominantly used to discriminate between speech and non-speech clusters in the TICC algorithm. Both of these cortical areas have already shown speech activity to various degrees in our prior publication [18]. Moreover, the plot suggest that the algorithm focused on a rather smaller subset of electrodes compared to our prior publication on synthesizing keywords where the supervised nVAD model based its decision on a much broader network of electrodes across motor, premotor and somatosensory cortices. We hypothesize that this is related to the different machine learning approaches (a RNN compared to the TICC algorithm), the increased number of word stimuli (50 stimuli instead of 6) and the variability in the data as some words in the 50-word corpus are longer and more effortful to articulate. Electrodes that show stronger variation in their activity patterns across words are therefore more likely to be treated as weaker indicators with respect to the voice activity binary classification task.

### 3.4. Predicting speech from neural activity

We compared all three classifiers with respect to two conditions: (1) trained on identified speech segments from the clustering approach, and (2) trained on ground truth speech activity like previous supervised nVAD studies. Here, we based our evaluation on a leave-one-day-out cross-validation method, where the test data is always isolated from every day in the training set to prevent day-specific information leaking which may wrongfully bias generalization. In every fold, we withheld another day from the training set as our validation data to verify proper model trainings. Note that the single day previously used in the development set to report intermediate results from the clustering approach was not used in this evaluation. For the LR and the convolutional network, we used context stacking up to 300 ms into the past as those classifiers would not otherwise model sequential dependencies.

**Figure 5.**
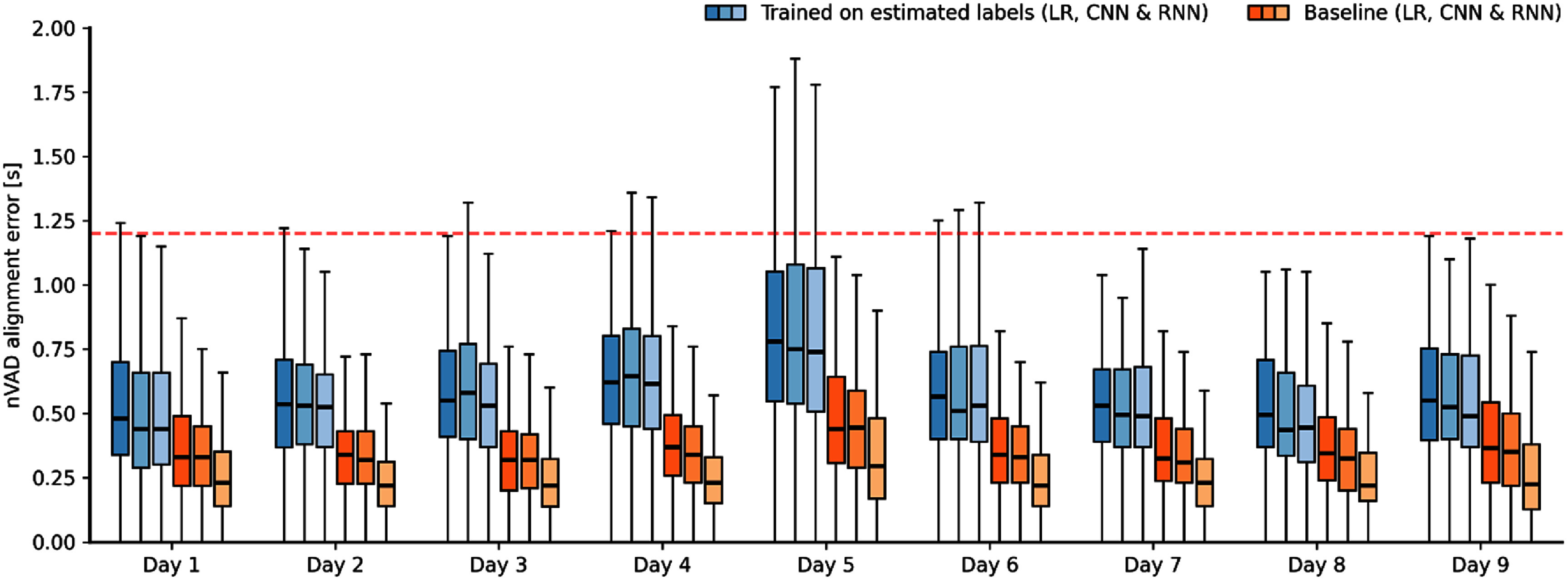
Cross validation results regarding the proposed approach and baseline models. Alignment errors are reported with respect to the specific held-out day in each fold. Box plots (samples per day: *n*_1_ = 306, *n*_2_ = 204, *n*_3_ = 204, *n*_4_ = 204, *n*_5_ = 204, *n*_6_ = 204, *n*_7_ = 204, *n*_8_ = 102, and *n*_9_ = 204) indicate that our approach achieves consistently higher error rates in the range of 140 and 215 ms than models trained on ground truth VAD information. Boxes represent data between the quartiles Q1 and Q2, while whiskers represent range within 1.5 times the interquartile range. The dashed red line shows average speaking duration.

Our results from the cross-validation are summarized in figure 5. For each day, we report the alignment errors for all three classifiers trained on estimated alignments from TICC (blue to light blue) and baseline models trained on ground truth speech information (orange to light orange) as boxplots (samples per day: *n*_1_ = 306, *n*_2_ = 204, *n*_3_ = 204, *n*_4_ = 204, *n*_5_ = 204, *n*_6_ = 204, *n*_7_ = 204, *n*_8_ = 102, and *n*_9_ = 204). The dashed red line indicates the average speaking duration of 1.2 s per prompted word from our participant. Across all days from the study period, we observed median error scores between 480 and 780 ms using the LR classifier, between 435 and 740 ms with our LeNet-like CNN, and between 440 and 740 ms with the RNN, when trained on estimated labels. Here, 50% of the trial-based errors were in the ranges of 400–770 ms, 390–760 ms, and 370–740 ms, respectively, for the corresponding classification method. Furthermore, we also observed that in 8% of the trials (146 out of 1836 trials) the RNN models were not capable of detecting speech instances at all or made prediction errors that exceeded the average speaking duration. For the CNN and LR models, those percentages were at 8.7% (160 trials) and 7.8% (144 trials), respectively. We excluded those outliers in figure 5.

Regarding the baseline methods, the RNN model achieved overall the lowest alignment errors with median scores between 220 and 295 ms across days, where 50% of the trials deviated between 140 and 350 ms from the ground truth acoustics. For the LR and the CNN, the alignment errors were higher between 320 and 440 ms, and between 310 and 445 ms in median, respectively. Across all three classifiers, 50% of the trials had alignment errors between 140 and 350 ms RNN, between 230 and 460 ms CNN, and between 230 and 500 ms LR. While RNNs trained on ground-truth data consistently achieved better performance compared to LR or CNN in the case of baseline models, this did not necessarily translate to the performance in the test set when trained on estimated labels from the clustering approach. Instead, model hyperparameters were optimized with respect to the validation set and therefore on labels found through TICC. Figure 5 indicates that all three classifiers achieved similar performance, suggesting that the misalignment between labels found through TICC and ground truth neglected individual model improvements.

We utilized a linear mixed-effects model (LMM) to quantify the impact of several factors on detection performance: (1) classification model type, (2) whether models were trained on ground truth data, and (3) interactions between model type and ground truth availability—accounting for random effects across days. Having the CNN model trained on estimated labels as a base case, we found that the LR appears to be inferior to the CNN model (+4 ms), while the RNN performs slightly better (−19 ms). However, *p*-values of 0.738 and 0.095 indicate that these differences are not significant. Instead, having the models trained on ground truth data led to highly significant (*p* < 0.001) improvements across all model types: −261 ms for LR, −280 ms for CNN and −355 ms for RNN. Moreover, the LMM also shows that RNNs significantly outperformed the other two classifiers when trained on ground truth data (*p* < 0.001). The day-specific standard deviation of 77.5 ms was relatively small compared to the residual standard deviation of 339 ms, indicating that day-to-day variations contribute only mildly to the overall variability in alignment errors.

Furthermore, we observed speech detection probability scores (mean ± standard deviation) of 0.79 ± 0.06 and 0.78 ± 0.07 for the RNN and LeNet models, respectively, as well as a score of 0.75 ± 0.07 for the LR across all days from our cross-validation analysis, indicating that frames corresponding to a speech segment have a high likelihood of being correctly classified as speech. With respect to false alarms, we achieved probability scores of 0.10 ± 0.03 for all model types and architectures, suggesting that the approach is not particularly prone to assigning non-speech frames incorrectly as speech. To put those values into perspective, we observed consistently higher speech detection probability scores of 0.89 ± 0.03 RNN, 0.84 ± 0.04 (LeNet), and 0.82 ± 0.03 LR from baseline models, leading to relative improvements of up to 13% compared to models trained on estimated labels by the TICC algorithm. Similarly, the false alarm probability was also lower with respective scores of 0.04 ± 0.01 RNN, 0.05 ± 0.01 CNN, and 0.05 ± 0.01 LR.

Although the results from our approach were not on par with baseline models trained on ground truth VAD information, we observed that our approach was still capable of detecting the majority of spoken speech, up to 91% per day, and on average 83% across days. To compute the majority detection rate, we determined how often more than 50% of the acoustic speech was assigned to the speech class, divided by the total amount of trials. This would be particularly useful for filtering out speech frames during online computations to obtain normalization statistics based on streaming neural activity. Please note that the results from the cross-validation analysis have been obtained through offline experiments. However, when deploying the models in our real-time system, we get identical classification results on every frame when streaming the same data from the test days—indicating that our approach achieves equal performance in real-time scenarios.

### 3.5. Generalizability towards unseen words

Next, we analyzed the applicability to spoken words beyond our training corpus of 50-word stimuli to quantify generalization. We recorded an additional corpus of 688 words (each word was only repeated once) across 7 sessions on one particular day (outside of the training days) and computed the mean alignment errors for all trials. The average speaking duration regarding of unseen words was 1.3 s per word. Our results do not show any substantial deviations from those word stimuli that were present in the training corpus. Given our three classification methods, the median alignment errors were between 440 and 480 ms RNN, between 450 and 510 ms CNN, and between 460 and 490 ms LR, where 50% of the trials occurred in ranges of 330 and 650 ms, between 350 and 650 ms, and 350–670 ms, respectively, suggesting that this approach is also applicable to unseen word stimuli. Although the reported ranges for the alignment errors on unseen words were slightly lower for unseen words when compared across all days from the cross-validation, this was primarily due to one day (D5) on which higher alignment errors occurred overall.

## 4. Discussion

Here, we demonstrate a BCI that is capable of identifying speech activity in real-time from ECoG signals recorded from speech-related cortical areas in a clinical trial participant living with ALS. Prior studies reporting on voice activity detection from neural activity have relied on ground truth acoustic speech information to train predictive models—a major challenge when translating such findings to paralyzed individuals who have lost their ability to speak. Our approach utilizes a graph-based clustering technique to localize consecutive segments in the neural data related to speech production. We designed an experiment paradigm that can infer which clusters most likely belong to speech activity based on their clustering lengths. By training three binary classification models on these estimated alignments, we were able to identify the majority of speech activity in up to 91% of the trials.

While the performance of those approaches was not on par with baseline models trained on ground truth acoustic speech information, it would not be reasonable to expect equivalent or better performance in the absence of ground truth. The timing and magnitude of muscular contractions preparing for and executing phonation and articulation do not have a one-to-one correspondence with the timing and magnitude of the acoustic waveform produced by speech [54], which serves as the ground truth for VAD. Consequently, the timing and magnitude of neural activity in sensorimotor cortex, which form the basis for nVAD, are not expected to be perfectly aligned with spoken acoustics. Moreover, while the signal-to-noise ratio of ECoG HG power modulation has proven sufficient for decoding speech, it is nevertheless non-stationary and dependent on imperfect estimates of its noise floor during non-speech segments, derived here from a separate session with cued speech segments. In spite of these challenges for nVAD, we found that our approach could detect the majority of speech. This is further supported by speech detection probability scores of up to 0.79, indicating that HG activity frames corresponding to speech production have a high likelihood for being detected as those. Analyses on seen and unseen word stimuli revealed that recall scores of approximately 79% could be achieved, compared to 93% from the RNN baseline models. While our current approach was not capable of always isolating each spoken word in its own unique segment, meaning that alignment errors can occur both outside and within the spoken word itself, additional postprocessing strategies may help combine neighboring segments that likely belong to the same word. Such strategies have been used in the past to correct misclassified frames based on a fixed window of predictions [45].

By interpreting and comparing cluster parameters, we found that assignments were mainly driven by differences of neural activity in a subset of the electrodes in the vM1 and dM1 cortical regions and their interconnections. In particular, we found in previous work [18] that electrodes in the lower part of the grid (vM1) consistently elicit strong HG responses during speech production tasks, while electrodes at the top of the grid have been observed with weaker responses. Our analysis suggests that the conditional dependencies between those electrodes provided information that the clustering algorithm predominantly selected for making class assignments. Even though many more electrodes show HG activations during overt speech production, the clustering approach converged to similar weights and interconnection weights for both speech and non-speech MRFs on those electrodes. One explanation of this behavior might lie in the variability of HG activity across word stimuli, and that the TICC algorithm identified those less reliably when making the binary assignment.

A limitation of our study is that our participant was still able to speak, albeit with significant dysarthria and poor intelligibility. Thus, it remains to be seen if our approach translates to patients who are incapable of producing audible speech. In this study, we focused intentionally on a patient who could still speak so that we could compare the performance of our approach with ground truth speech acoustics and to estimate the extent of alignment errors—which would not have been possible if speech had been absent.

In this pilot study, we addressed the open challenge of training a BCI that identifies speech without having time-aligned neural and acoustic data. Our results show that a graph-based clustering approach can identify segments of spoken speech in neural recordings with median alignment mismatches below 500 ms. Despite this inaccuracy, we were able to train VAD models and deploy them in a real-time streaming scenario to predict speech activity online. The error rate may be small enough for practical application. We believe this would be particularly useful for avoiding the inclusion of speech frames when calculating baseline neural activity during non-speech segments and for real-time gating of speech decoders in speech BCIs, including brain-to-text and brain-to-speech applications. Moreover, our approach could also benefit BCI systems by acting as a switch to toggle on the decoder when the user generates silent speech, and toggle off after some time of silence. This would prevent undesired random speech decoding when the user is doing other tasks that somehow affect motor activity. The low false alarm probabilities suggest that undesired toggling of such a switch would not be very likely. Future work is necessary to determine whether our approach is equally effective for individuals who can no longer produce audible speech.

## Data Availability

The data that support the findings of the study are openly available at the following URL/DOI: https://osf.io/s9juc/. Neural recordings are prepared in the MATLAB file format version 5, where time-aligned anonymized acoustic speech is stored in the wav file format.

## Code availability

All source code supporting this study will be made publicly available on https://github.com/cronelab/corticom-neural-vad upon acceptance of the manuscript. Moreover, the repository also comes with a bash script which can be used to replicate all steps done in this study, including rendering the figures and running the real-time BCI on streamed signals.
